# Chemical Composition, Insecticidal and Mosquito Larvicidal Activities of Allspice (*Pimenta dioica*) Essential Oil

**DOI:** 10.3390/molecules26216698

**Published:** 2021-11-05

**Authors:** Arunaksharan Narayanankutty, Aswathi Moothakoottil Kuttithodi, Ahmed Alfarhan, Rajakrishnan Rajagopal, Damia Barcelo

**Affiliations:** 1 Division of Cell and Molecular Biology, PG and Research Department of Zoology, St. Joseph’s College (Autonomous), Devagiri, Calicut 673008, India; abcaswathi@gmail.com; 2 Department of Botany and Microbiology, College of Science, King Saud University, P.O. Box 2455, Riyadh 11451, Saudi Arabia; rrajagopal@ksu.edu.sa; 3 Water and Soil Research Group, Department of Environmental Chemistry, IDAEA-CSIC, JORDI GIRONA 18-26, 08034 Barcelona, Spain; damia.barcelo@idaea.csic.es

**Keywords:** *Pimenta dioica* essential oil, larvicidal property, biopesticidal activity, *Sitophilus oryzae*, *Callosobruchus maculatus*

## Abstract

Essential oils are biologically and environmentally safe pesticidal compounds yielded from aromatic plants. Spices are important sources of essential oils, and they are widely used in the medicine, food, and various other industries. Among the different spices, Allspice (*Pimenta dioica*) is underexplored in terms of its biological efficacy and a limited number of studies are available on the chemical composition of Allspice essential oil (AEO); thus, the present study evaluated the larvicidal property, the repellency, and the fumigant toxicity against common pests of stored products of AEO. AEO was found to inhibit the survival of larvae of such vectors as *Aedis, Culex*, and *Armigeres* species. Further, AEO was found to exert repellant effects against the pests of such stored products as *Sitophilus, Callosobruchus,* and *Tribolium*. Similarly, the fumigant toxicity was found to be high for AEO against these species. The contact toxicity of AEO was high against *Sitophilus* and *Callosobruchus*. Apart from that, the essential oil was found to be safe against a non-target organism (guppy fishes) and was found to be non-genotoxic in an *Allium cepa* model. Overall, the results of the present study indicate that the essential oil from Allspice could be used as an environmentally safe larvicidal and biopesticidal compound.

## 1. Introduction

Insects are important organisms that are known to be beneficial to humans in many respects, including nutrition, agriculture, and environmental stability. In contrast, several other insects are harmful in terms of health damage, crop damage, or even causing pollution. Among these, pests and vectors are the most destructive organisms and cause significant health damages and productivity losses. Insect pests cause severe damage to crops and products by feeding on the plant’s parts or the stored cereals and pulses. *Sitophilus* sp., *Tribolium* sp., and *Callosobruchus* sp. are the most common insect pests of stored food materials such as cereals, grains, and pulses. Increased attacks from these pests often result in reduced productivity and, subsequently, a diminished economy [1]. Outbreaks of these pests often threaten global food security and, thereby, produce health concerns [2]. Apart from pests, vectors are another important group of insects comprised of mosquitos, ticks, and mites that have a significant impact on healthcare systems. Mosquitoes act as the prime vectors for the transmission of such pathogens as Zika virus, *Plasmodium* sp. (a malarial parasite), chikungunya virus, and dengue virus. The spread of mosquito-borne disease causes more than 400,000 deaths annually [3].

Taking into consideration the economic and agricultural losses caused by the different pests and the health risk posed by vectors, it is necessary to control vector and pest populations. Classical control measures, such as the application of an insecticide, have been capable of maintaining the population of these insects in check. Gradually, however, various insects gained resistance to these insecticides, thereby limiting the efficacy of control measures [4,5]. Apart from the limited efficacy, the environmental issues caused by insecticides and pest control agents constitute another emerging concern [6]. The various classes of pesticides have been proven to be phytotoxic to plants and also to the agriculture workers involved in their application [7,8]. In addition, cancer and neurotoxic effects are also common among individuals with chronic exposure to these chemical agents [9]. All this has led to an increase in the demand for biologically and environmentally safe molecules that can be used as pest-control and vector-control agents.

Essential oils have emerged as important agents that are capable of repelling and eliminating a variety of pests and vectors of human diseases. Essential oils extracted from traditionally used spices have a peculiar aroma and are reported to be superior to other essential oils from non-spice plants. It has therefore been suggested that integrated pest management techniques employing plant-derived essential oils could be a successful alternative to chemical pesticides and provide a better measure for pest control [10]. *Pimenta dioica* is such a spice plant and has been reported to have several health-promoting effects [11]. The preliminary studies by Rocha Voris et al. [12] have indicated the possible use of essential oil from *P. dioica* fruits against larvae and adults of Aedes sp. Furthermore, the essential oil was also found to inhibit the growth of *Aspergillus* and the production of alfatoxins by them [13]. It has also been reported that this essential oil acts as an antibacterial agent and was found to inhibit inflammation and edema conditions [14,15]. However, the essential oils of *P. dioica* leaves have yet to be evaluated in terms of their larvicidal and insecticidal potential; furthermore, no reports are available on their environmental toxicity. Hence, the assumption is that, by virtue of the bioactive components present, the Allspice (*P. dioica*) essential oil (AEO) may exert significant growth inhibitory effects on mosquito larvae and common insect pests. Thus, this study evaluated the application of AEO in the control of mosquito larval and pest populations. In addition, the effect of AEO on non-targeted species, phytotoxicity, and an *Allium cepa* model of genotoxicity was also evaluated.

## 2. Results

### 2.1. Average Yield of AEO and Its Chemical Characterization

The Allspice essential oil obtained in this study was 1.02 ± 0.08% in each of three independent preparations. Further, it was chemically characterized using GC-MS techniques. Eugenol (65.82%) and its derivative methyl eugenol (15.22%) were predominant in the essential oil. Additionally, caryophyllene, cineole, humulene, and terpinolene were observed in the Allspice essential oil (Table 1).

### 2.2. Lethality of AEO on the Different Mosquito Larval Forms

**Figure 1:**
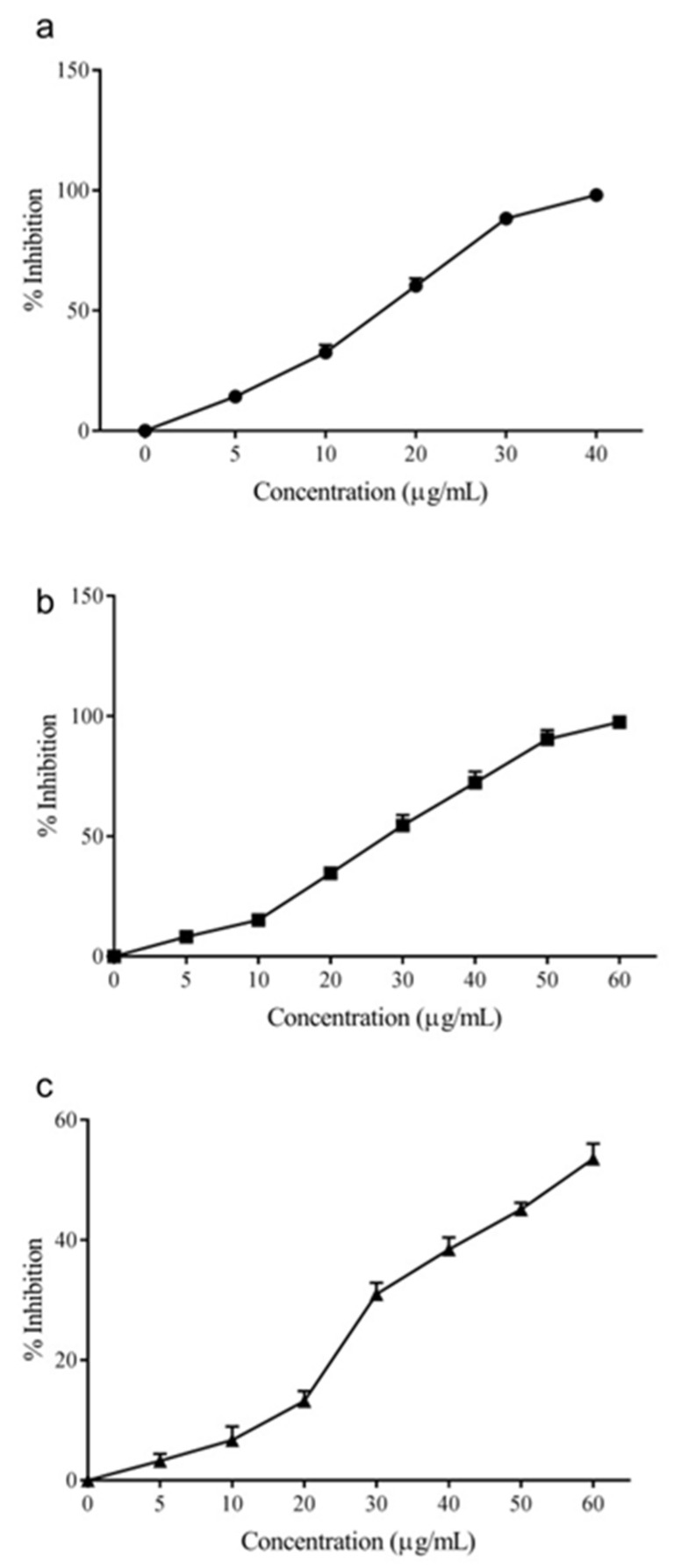
The larvicidal activity of Allspice essential oil against *Aedes aegypti* (**a**), *Culex quinquefasciatus* (**b**), and *Armigeres subalbatus* (**c**) larvae over 24 h.

The larvicidal activity of AEO over 24 h was found to be high against *Aedes aegypti* with a LC_50_ value of 18.5 ± 1.2 µg/mL (Figure 1). Similarly, AEO was found to be effective against *Culex quinquefasciatus* and *Armigeres subalbatus* species, with respective LC_50_ values of 28.9 ± 1.6 and 55.1 ± 3.1 µg/mL. Among these, *Aedes aegypti* was the most sensitive to AEO, whereas the larvae of *A. subalbatus* were the most resistant. The IC_50_ values for 24 h and 48 h are listed in Supplementary Material Table S1.

### 2.3. Potential Role of the Allspice Essential Oil in Eliminating Pests

AEO was found to have anti-feedant activity against *Sitophilus* species (1.57 ± 0.09 µg/g of wheat flour). Likewise, the respective IC_50_ value against *T. castaneum* and *C. maculatus* was 2.04 ± 0.10 µg/g and 1.88 ± 0.06 µg/g (Table 2). In addition, significant repellant activity was also observed for AEO against the three pest species, the highest being against *S. oryzae* (3.79 ± 0.18 µg/L of air). Supplementary Material Table S2 shows a comparison of the efficacy of a single dose of AEO against different pests for each level of biological activity.

The lethality of AEO was determined in terms of its fumigant potential as well as its contact toxicity. The respective IC_50_ values are shown in Table 2. AEO was the most effective against *S. oryzae* compared with the other pests.

### 2.4. Ecological Safety Analysis of AEO

The ecological safety analysis of AEO was performed in terms of phytotoxicity, genotoxicity, and toxic effects on non-targeted organisms. As indicated in Table 3, we observed no significant variation in the germination potential of grains treated with AEO doses. Further, the treatment with AEO was found to remain non-toxic towards *A. cepa* mitotic events, and we did not observe any chromosomal damage to the tissue, even in *A. cepa* cells treated with the highest dose of AEO (Table 4). Similarly, the essential oil was not observed to induce any kind of behavioral changes in guppy fishes over a period of 48 h (Table 5) and at different time points (Supplementary Material Table S3). Hence, the results confirm that Allspice essential oil is safe in terms of germination potential, mitotic cell division, and non-targeted organism toxicity.

## 3. Discussion

Stress volatiles from plants are important chemicals known to be highly active in different aspects of biological assays. A considerable number of these stress volatiles are essential oils. These essential oils are known to have antimicrobial, anti-edematous, antioxidant, anti-proliferative, and antidiabetic activities. However, the most important attribute of an essential oil is its ability to regulate insect and larval populations. This study therefore evaluated the ability of a well-known spice—Allspice (*P. dioica*)—to eliminate the larvae of different mosquito species as well as insect pests in stored food materials.

The results indicate the presence of eugenol and its derivative in the essential oil of Allspice leaves. Furthermore, molecules such as caryophyllene and cineole were identified in the AEO. Previous studies by Rocha Voris et al. [12] and Chaudhari et al. [13] also found a large amount of eugenol in the essential oil extracted from buds and bark of Allspice. These studies were also indicative of the larvicidal properties of the essential oil from Allspice. Supporting their findings, we also observed significant larvicidal activity against the various mosquito species, and AEO exhibited the highest activity against the *Aedes* species. *Aedes* species are distributed globally and have been reported to act as vectors of arboviral diseases [16], Chikungunya virus [17], dengue virus [18], and the recent Zika viral diseases [19]. Therefore, controlling the larval population of *Aedes* species by AEO may help to prevent the spread of these diseases considerably. It has previously been reported that eugenol and its derivatives have significant larvicidal properties against a wide variety of mosquitoes [20,21]. Furthermore, the application of pesticides to control larval populations has raised concerns about non-targeted organism toxicities; however, the present study confirmed the non-toxic nature of AEO against guppy fishes as a model. Hence, this result suggests that AEO is a potential larvicidal candidate with low non-targeted organism toxicity, which makes it preferable over synthetic and chemical insecticides.

In connection with these, AEO has been found to be effective as an anti-feedant and a repellent agent against different pests that are common in stored cereals and pulses. These pests are known to cause significant losses in productivity and to the economy by damaging the stored food materials. Hence, the control of their population by AEO has great potential to improve the storage quality of food materials. In addition, AEO was found to significantly increase the mortality rate in these pests as a fumigant and contact insecticide. The fumigant potential is more influential as it can reach even the smaller areas of storage packets or storage houses. The predominant compound in AEO, eugenol, has been shown to exert an anti-feedant effect on the red palm weevil [22]; likewise, it was found to be a strong insecticide against *Sitophilus* species [23]. Recent studies have also indicated that synthetic derivatives of eugenol can be an alternative to chemical insecticides [24].

In addition to these findings, the study also confirmed the safety of AEO. It was observed that AEO is not toxic to germinating seeds, even at its highest doses. This widens the range of possible uses of AEO as an insecticide, even for areas of seed storage. Furthermore, there was no sign of genotoxic activity in the *A. cepa* model analyzed. Overall, AEO could be a suitable non-toxic or ecologically safe alternative to chemical pesticides.

## 4. Materials and Methods

### 4.1. Collection, Extraction, and Chemical Composition Analysis of Allspice (P. dioica) Leaf Essential Oil

*Pimenta dioica* leaves (other than tender leaves) were collected from the Kozhikode district during the August–September period and authenticated by Dr. Anisha M Sathyan, Botanist, Malabar Christian College, Kerala, India. The leaves were cleaned to remove the dust, refrigerated at −80 °C (Remi, Mumbai, India), and powdered using a mortar. A hydro-distillation technique was employed for the extraction of essential oil. The extraction was carried out in three independent isolations and the yield and composition of each were recorded. The extraction procedure and storage were carried out according to the methods described by Chaverri and Cicció [25] without significant modifications.

The chemical nature of the obtained essential oil was determined by the gas chromatography–mass spectroscopy (Shimadzu, Kyoto, Japan) technique described in the previous published reports of Lorenzo-Leal, Palou, and López-Malo [26]. The chemical nature was predicted from the chromatogram using the retention index and the *m*/*z* ratio and by comparison with the standard NIST library.

### 4.2. Efficacy of Pimenta dioica Essential Oil against Mosquito Larvae

The mosquito larvicidal activity was determined according to the methods described by Hung et al. [27]. The mosquito species selected for the study included *Aedes aegypti, Culex quinquefasciatus,* and *Armigeres subalbatus* species. The mosquitoes were grown in separate insect cages for 10 generations. The larvae of each species were reared and were collected at their third instar state for experimental purposes. About 50 larvae of each mosquito species were placed in 500 mL beakers and varying doses of the essential oil were dissolved in them. The larvae in each bottle were observed for 24 h and the mortality rate was recorded on time. The percentage mortality of AEO was determined by comparison to a normal control at the end of a 24-h period.

### 4.3. Allspice Essential Oil (AEO) and Pest Control Capacity

#### 4.3.1. Anti-Feedant Activity of AEO on Wheat Flour

The anti-feedant activity of AEO was determined according to the methods described by Zhang et al. [28]. The percentage inhibition of wheat flour intake was estimated by comparing the consumption of an AEO and wheat flour mixture with that of wheat flour without AEO. For the anti-feedant assay, the sample size used per group was 25 pests.

#### 4.3.2. Repellant Activity

The repellant potential of essential oils is important to the management of pests. This study employed the protocol discussed by Patiño-Bayona et al. [20]. The repellency was estimated as the ability of AEO to displace the insects from an essential-oil-exposed bottle to an untreated bottle and the median repellant dose is expressed as µg/L of air. Twenty-five pests per group were selected for the repellency assays.

#### 4.3.3. AEO as A Potential Fumigant Agent

The toxicity of AEO as a fumigant agent against the different pests was estimated by the protocols laid down by Patiño-Bayona et al. [29]. The activity was determined as the lethal dose of AEO as a fumigant and is expressed as the LC_50_ value in µg/L of air. The fumigant potential was assessed using a sample size of 25 pests per treatment group.

#### 4.3.4. Determination of AEO’s Contact Toxicity

AEO at different doses was applied to adult insects in their pro-thorax region according to the methods of Paventi et al. [30]. The untreated and essential-oil-treated insects were observed for 48 h continuously, and the lethality and lethal concentration (the LD_50_ value) were determined. For the contact toxicity tests, 50 pests were chosen per treatment group.

### 4.4. Allspice Essential Oil and Its Effect on the Germination Potential of Grains

The effect of AEO on grain seed germination is considered to be an indicator of its phytotoxicity. The analysis was carried out as per the studies of Ibáñez and Blázquez [31]. The wheat grains (20 nos) were allowed to germinate in the presence and absence of AEO for 6 days. Protrusion of a well-developed radicle from the grain with a length of 1 cm was considered to be an indication of germination.

### 4.5. Non-Targeted Species Toxicity in Guppy Fish (Poecilia reticulata)

The guppy fish (*Poecilia reticulata*) is widely known for its insectivorous nature, especially with respect to the larvae of mosquitos. Hence, these fish are threatened during the application of a toxic agent against mosquito larvae. So, the species is an apt non-target organism. Fishes (with a length of 3.05 ± 0.11 cm and a body weight of 1.08 ± 0.09 g) were exposed to different doses of AEO for 48 h and observed continuously for signs of toxicity [32].

### 4.6. AEO’s Genotoxic Effect on an Allium Cepa Model of Mitotic Damage

*A. cepa* bulbs with a uniform weight were selected and planted in individually compartmentalized plates and allowed to grow in the presence of varying concentrations of AEO for 72 h. A set of compartments were left untreated and were considered a control. At the end of the 72-h period, the actively dividing portions of each onion bulb were collected and fixed, and a smear was prepared [33]. The mitotic index, sticky and bridged chromosomes, and the presence of micronuclei were analyzed microscopically.

### 4.7. Statistical Analysis

The essential oil’s preparation was carried out three times and the mean and SD of the three independent experiments are presented. All other bioassays were repeated five times. The statistical tool employed was one-way ANOVA followed by Tukey’s test using GraphPad Prism version 7.00 (La Jolla, CA, USA).

## 5. Conclusions

The Allspice (*P. dioica*) leaf essential oil was found to be beneficial in the prevention of the growth of mosquito larvae and to thereby be a promising candidate for mosquito eradication. Furthermore, we observed AEO to have significant anti-feedant and repellent abilities, which again emphasize its possible pest-repellent properties. Lethality to different pests was also observed, suggesting that AEO could be used as a fumigant and contact insecticide. These results suggest the use of AEO for the possible elimination of pests by repelling as well as by killing pests. In addition, the essential oil was also found to be safe towards germinating grains, cellular division, the genome, and non-targeted animal species. We therefore conclude that AEO could be an ecologically safe insecticide and larvicide.

## Figures and Tables

**Table 1:** GC-MS analysis of the essential oil extracted from *Pimenta dioica* leaves using steam distillation.

Sl. No.	Compound	Retention Index	% ^a^
1	*α*-Thujene	937	0.11
2	*α*-Pinene	946	0.06
3	*β*-Pinene	986	0.15
4	Myrcene	991	0.44
5	*α*-Phellandrene	1005	1.46
6	*α*-Terpinene	1020	0.08
7	*p*-Cymene	1026	0.66
8	Ocimene	1030	0.22
9	1,8-Cineole	1033	1.86
10	*γ*-Terpinene	1062	0.40
11	Terpinolene	1092	1.05
12	*cis*-Sabinene hydrate	1097	0.12
13	Linalool	1098	0.05
14	*trans*-*p*-Menth-2,8-dien1-ol	1108	0.10
15	*cis-p*-Mentha-2,8-dien1-ol	1120	0.01
16	*β*-Terpineol	1180	0.66
17	*p*-Cymene-1-ol	1185	0.18
18	Citronellol	1220	0.46
19	Linalyl acetate	1250	0.01
20	Terpinyl acetate	1333	0.61
21	Eugenol	1351	65.82
22	Methyl eugenol	1401	15.22
23	Caryophyllene	1428	4.03
24	*β*-Gurjunene	1432	0.04
25	Aromadendrene	1440	0.10
26	Alloaromadendrene	1461	0.01
27	*α*-Humulene	1465	1.58
28	Cedrene	1468	0.24
29	Bergamotene	1470	0.06
30	*γ*-Muurolene	1475	0.84
31	Patchulene	1486	0.24
32	Germacrene D	1490	0.49
33	*β*-Bisabolene	1506	0.10
34	Caryophyllene oxide	1566	0.61

(^a^ Relative area = relative content expressed as a percentage of the total oil composition).

**Table 2:** The insecticidal properties of *Pimenta dioica* (Allspice) essential oil extracted by steam distillation estimated in terms of repellent efficacy, contact toxicity, and fumigant toxicity.

Test	Assay	IC_50_ Values
Anti-feedant assay (IC_50_ µg/g wheat flour)	*Sitophilus oryzae*	1.57 ± 0.09
	*Tribolium castaneum*	2.04 ± 0.10
	*Callosobruchus maculatus*	1.88 ± 0.06
Repellent activity RC_50_ (µg/L of air)	*Sitophilus oryzae*	3.79 ± 0.18
	*Tribolium castaneum*	5.38 ± 0.22
	*Callosobruchus maculatus*	5.15 ± 0.31
Fumigant toxicity LC_50_ (µg/L of air)	*Sitophilus oryzae*	14.5 ± 0.61
	*Tribolium castaneum*	19.1 ± 0.43
	*Callosobruchus maculatus*	18.5 ± 0.67
Contact toxicity LD_50_ (µg/mm^2^)	*Sitophilus oryzae*	75.1 ± 3.08
	*Tribolium castaneum*	81.6 ± 2.04
	*Callosobruchus maculatus*	69.3 ± 1.55

**Table 3:** Phytotoxic effect of Allspice (*Pimenta dioica)* essential oil in terms of the germination potential of wheat (% germination).

Duration of Exposure in Hours	Untreated Grains	*Pimenta dioica* Essential Oil (µg/mL)		
		100	250	500
48	14.1 ± 2.4	13.8 ± 1.4	13.4 ± 1.2	13.5 ± 2.0
72	32.6 ± 1.3	33.4 ± 1.6	32.1 ± 1.5	32.9 ± 1.1
96	60.2 ± 2.4	58.6± 2.1	58.4 ± 2.4	59.1 ± 1.9
120	82.1 ± 3.2	80.6 ± 3.0	79.2 ± 3.1	80.5 ± 2.4
144	92.6 ± 1.1	90.2 ± 3.3	91.3 ± 2.4	91.4 ± 2.3

**Table 4:** Genotoxicity assay of Allspice essential oil (AEO) in model *Allium cepa* mitotic cells.

Treatment Group	Mitotic Index (%)	Frequency of Aberrant Cells (%)
Normal (Untreated)	12.56 ± 0.32	0.42 ± 0.03
AEO (1 mg/mL)	12.41 ± 0.17	0.50 ± 0.05
AEO (2.5 mg/mL)	11.88 ± 0.41	0.51 ± 0.07
AEO (5 mg/mL)	12.01 ± 0.40	0.48 ± 0.03
AEO (10 mg/mL)	12.37 ± 0.22	0.51 ± 0.08

**Table 5:** Toxicity analysis of Allspice essential oil (AEO) against a non-targeted organism (guppy fishes) at different doses.

Treatment Group	% Mortality	Fishes Having Difficulty Swimming	Fishes with a Color Change	Time Spent on Top of the Water (Seconds)
Normal	0	0	0	31.2 ± 4.0
AEO (50 µg/mL)	0	0	0	37.6 ± 2.0
AEO (100 µg/mL)	0	0	0	38.3 ± 4.0
AEO (200 µg/mL)	0	0	0	30.5 ± 5.0
AEO (250 µg/mL)	0	0	0	34.2 ± 4.0

## Data Availability

The data may be shared upon a valid request.

## Supplementary Materials

The following are available online. Table S1: Larvicidal potential of Allspice (*Pimenta dioica*) essential oil at different time points of treatment; the efficacy has been expressed as the half-maximal lethal concentration (LC_50_) (μg/mL), Table S2: Insecticidal property of Allspice essential oil at a specific dose over different time points on insects pests, Table S3: Effect of Allspice essential oil against non-target organism at varying doses over different time periods.

## Sample Availability

Samples of the compounds are available from the corresponding author.
