# Supplementary material for: Chemical Composition, Insecticidal and Mosquito Larvicidal Activities of Allspice (Pimenta dioica) Essential Oil
Source: Molecules. 2021 Nov 5;26(21):6698. doi: 10.3390/molecules26216698 (PMC8588154; doi:10.3390/molecules26216698)
Supplement: Supplementary file 1 [file molecules-26-06698-s001.zip › molecules-1405887-supplementary.pdf]

Supplementary Material Table S1. Larvicidal potential of Allspice (*Pimenta dioica*) essential oil at different time points of treatment; the efficacy has been expressed as the half-maximal lethal concentration (LC50) (μg/mL)

**Mosquito**	**LC50** (μg/mL)	
	**24 hour**	**48 hour**
Armigeres subalbatus	55.1±3.1	37.61± 2.4
Aedes aegypti	18.5±1.2	11.03± 1.3
Culex tritaeniorhynchus	28.9±1.6	19.8± 2.7

Supplementary Material Table S2. Insecticidal property of Allspice essential oil at a specific dose over different time points on insects pests

Test	Assay	Percentage inhibition
Anti-feedant assay (1.50 μg/g wheat flour)	*Sitophilus oryzae*	46.92± 3.14
	Tribolium castaneum	32.57± 3.06
	Callosobruchus maculatus	39.34± 2.78
Repellent activity (5.0 μg/L of air)	*Sitophilus oryzae*	72.44± 4.81
	Tribolium castaneum	43.09± 4.17
	Callosobruchus maculatus	45.62± 3.41
Fumigant toxicity (15.0 μg/L of air)	*Sitophilus oryzae*	54.90± 4.11
	Tribolium castaneum	36.73± 4.48
	Callosobruchus maculatus	42.27± 2.88
Contact toxicity (70.0 $\mu g/mm^2$)	*Sitophilus oryzae*	46.29± 4.06
	Tribolium castaneum	38.73± 3.75
	Callosobruchus maculatus	52.90± 2.82

Supplementary Material Table S3. Effect of Allspice essential oil against non-target organism at varying doses over different time periods

Treatment group	% Mortality			Abnormal behaviors		
	24 h	48 h	72 h	24 h	48 h	72 h
Normal	0	0	0	-	-	-
AEO (50 µg/mL)	0	0	0	-	-	-
AEO (100 µg/mL)	0	0	0	-	-	-
AEO (200 µg/mL)	0	0	0	-	-	Mild
AEO (250 µg/mL)	0	0	1	-	-	Mild
